# Too much medicine? Scientific and ethical issues from a comparison between two conflicting paradigms

**DOI:** 10.1186/s12889-019-6442-9

**Published:** 2019-01-22

**Authors:** Francesco Attena

**Affiliations:** Department of Experimental Medicine, School of Medicine, University of Campania, Via Luciano Armanni 5, 80138 Naples, Italy

**Keywords:** Systems medicine, Harm-benefit assessment, Breast cancer screening, Too much medicine, Paradigm, Incommensurability, Ethics

## Abstract

**Background:**

The role of medicine in society appears to be focused on two views, which may be summarized as follows: “Doing more means doing better” (paradigm A) and “Doing more does not mean doing better” (paradigm B).

**Main body:**

I compared paradigms A and B both in terms of a single clinical condition and in the general context of a medical system. For a single clinical condition, I analyzed breast cancer screening. There are at least seven interconnected issues that influence the conflict between paradigms A and B in the debate on breast cancer screening: disconnection between research and practice; scarcity of information given to women; how “political correctness” can influence the choice of a health policy; professional interests; doubts about effectiveness; incommensurability between harms and benefits; and the difficulty in making dichotomous decisions with discrete variables. As a general approach to medicine, the main representative of paradigm A is systems medicine. As representatives of paradigm B, I identified the following approaches or movements: choosing wisely; watchful waiting; the Too Much Medicine campaign; slow medicine; complaints against overdiagnosis; and quaternary prevention. I showed that both as a single condition and as a general approach to medicine, the comparison was entirely reducible to a harm-benefit analysis; moreover, in both cases, the two paradigms are in many respects incommensurable. This transfers the debate to the ethical level; consequently, scientists and the public have equal rights and competence to debate on this subject. Moreover, systems medicine has many ethical problems that could limit its spread.

**Conclusion:**

I made some hypotheses about scenarios for the future of medicine. I particularly focused on whether systems medicine would become increasingly accessible and widespread in the population or whether it would be downsized because its promises have not been maintained or ethical problems will become unsustainable.

## Background

Current tendencies regarding the role of medicine in society and about the extent of interventions for a population appear to be focused on two opposite views, or paradigms. Those paradigms may be summarized as follows. According to paradigm A (PA), “Doing more means doing better.” Conversely, according to paradigm B (PB), “Doing more does not mean doing better.”

In the past, these two views have had their various advocates. Against the excess of ineffective treatments, the movement of therapeutic nihilism took place at the end of the nineteenth century. However, in the early twentieth century, doctors had to choose between Galenic methods (purges, emetics, and bloodletting) and the Hippocratic method (wait, observe, console) [1]. Currently, the highest expression of PA is found in systems biology and systems medicine [2]. By contrast, PB is represented by various approaches and movements that oppose the excesses of modern medicine. The propensity toward PA or PB is also an attitude of individual health operators in terms of being more or less interventionist. The propensity may also be a cultural feature of particular nations; for example, the United States is a typical PA country.

Since its origins, modern medicine has become progressively more invasive into people’s lives and into society in general in pursuit of its goal of always seeking to improve. Beyond the purpose of doing more and doing something better, the main reasons for such developments are well known. From the perspective of the citizen, the reasons are as follows: improving the quality of life; the obsessive search for well-being; unrealistic expectations about the ability of medicine to solve all health problems; health anxiety (i.e. the distress, anxiety or sensation of serious illness, often unfounded, that a person feels regarding his/her personal health); the changed doctor-patient relationship from a paternalistic to an equal one. From the medical viewpoint, these new attitudes have led to greater medical-legal disputes and the birth of defensive medicine. Lastly, health industries foster health consumerism for the purpose of pursuing profits through increasingly expensive drugs and constant technological innovation. All these modern conditions are closely related and lead (both in the clinic and in terms of prevention, both diagnostically and therapeutically) toward medical interventionism rather than non-interventionism.

At the individual level, the best-known consequences of this hyper-interventionism have been overdiagnosis and overtreatment [3]; at the social level, the consequences have been the concept of medicalization in the negative sense and the problem of economic unsustainability of public health systems. These negative consequences and the anomalies of Kuhnian language have given rise to PB following the classic scheme of a paradigm shift [4].

The present study makes a comparison between PA and PB. It mainly does so in terms of clinical practice and preventive diagnosis: it adopts this approach because early diagnosis underscores the main differences between the two paradigms. Moreover, the two paradigms are compared with respect to a single clinical condition and with the general medical system. Maintaining Kuhnian terminology, I apply the concept of incommensurability in both situations. Incommensurability is deeper and better suited to the needs of the present discussion than other more generic concepts, such as incomparability, dissimilarity, and divergence. I are aware that the rigid opposition between these two points of view is somewhat artificial, the reality is more nuanced than the two paradigms indicate. Therefore, this simplification allow us to formulate a more linear and comprehensible reasoning.

## Incommensurability

Incommensurability has different meanings. In mathematics, it signifies that items cannot be precisely measured and compared using some common scale of unit of values. However, in the present study, I use the term in a way similar to the Kuhnian sense. In that sense, scientific theories are incommensurable if scientists cannot discuss them using a shared nomenclature that allows direct comparison of theories to determine which is more valid or useful. The reason for that lack of comparison is that the theories belong to two different scientific paradigms [4].

In this study and following the above definition, I use the term “incommensurability” in two ways: with respect to a single disease and with respect to the entire medical system.

A) *Incommensurability of harm-benefit assessment*. This occurs when the harms and benefits for a particular disease affect different domains of health status [5], which generates a conflict between PA and PB. Of course, I are aware that there are many conditions in which the harms and benefits can be compared through a common denominator (for example, mortality, quality-adjusted life-years); however, I believe that the incommensurability of harms and benefits is an underestimated topic, and so I addressed the following condition.

B) *Incommensurability between paradigms*. In the first instance, I assumed that PA and PB ignore each other: they do not have a common language and do not try to work together to find a shared solution. Ultimately, a comparison between PA and PB is entirely reducible to a (meta-) assessment between the harms and benefits.

I begin my analysis with an examination of a single disease: I present, as an example, the debate about breast cancer screening. I then investigate PA and PB in the general context of medical systems.

## Incommensurability of harm-benefit assessment

### Debate about breast cancer screening

Breast cancer screening is a good example of a conflict between two different approaches to screening: PA favors performing it; PB does not. Moreover, breast cancer screening is particularly interesting because it is a much-debated topic. There are at least seven interconnected issues that influence the conflict between PA and PB: disconnection between research and practice; scarcity of information given to women; how “political correctness” can influence the choice of a health policy; professional interests; doubts about effectiveness; incommensurability between harms and benefits; and the difficulty in making dichotomous decisions with continuous variables.

The first four items are not strictly scientific issues with respect to PA. Unlike with the many doubts highlighted by scientific debate, the benefits and effectiveness of screening are still overestimated in clinical practice [6–8]. For many health operators, the difficulties include updating procedures (disconnection between research and practice); at the population level, the information to women is still partly biased toward screening (scarcity of information given to women) [9, 10]. In many countries (and in my experience, it is certainly the case in Italy), it is not possible to speak against screening; according to [11], even some scientists have sacrificed sound scientific principles to arrive at politically acceptable results. Finally, many health operators can distort the judgment about the utility of screening owing to their particular interests in the field (e.g., as radiologists, surgeons, breast specialists) [12].

From a scientific perspective (the final three items in the above list) the debate about breast cancer screening is intense and engaged on two levels. The first level concerns effectiveness: beside the great amount of studies which have summarized the effectiveness of breast cancer screening [13–17], other studies have strongly questioned the ability of screening to reduce mortality [18–22]. If the effectiveness is accepted, the second level of discussion concerns the harm-benefit ratio: on the one hand, prevention of breast cancer mortality, and on the other hand, diagnostic anticipation, false positives, false positives after biopsy, and overdiagnosis [23–27].

The Canadian Task Force calculated that in the age-group of 40–49 years for each death from breast cancer prevented, it is necessary to screen 2108 women for 11 years, with 690 false positives and 75 false positives after biopsy. For the age-range of 50–69 years, the figures are 721 women, 204 false positives, and 26 false positives after biopsy [28]. Overdiagnosis is a recent, controversial issue owing to the difficulties involves in calculation [29]. The Independent UK Panel on Breast Cancer Screening calculated that for each death from breast cancer prevented, there are three overdiagnoses [13]. A more recent study investigated age-specific overdiagnosis for ductal carcinoma in situ and invasive breast cancer; it obtained a wide range of results: less than 1% among 40-year-old women with a screen detected cancer, up to 30% at age 80 years for ductal carcinoma in situ [30].

The US Preventive Service Task Force (USPSTF) recommends biennial screening mammography for women aged 50–74 years as a B recommendation. The task force makes that recommendation using a rather convoluted statement: “The USPSTF recommends the service. There is high certainty that the net benefit is moderate or there is moderate certainty that the net benefit is moderate to substantial” [16].

#### Incommensurability and undecidability

It is necessary to determine what these data tell us about the harm-benefit ratio. From a scientific viewpoint, the data are not very enlightening owing to the difficult in assessing that ratio. From an ethical perspective, an insoluble dilemma results. The dilemma arises mainly because in this example, harms and benefits are incommensurable. First, they belong to substantially different domains [5], i.e., preventing death versus various harms. Second, regarding the difficulty in making dichotomous decisions on continuous variables, that means (simplifying the argument), how many overdiagnoses (or, e.g., false positives) are acceptable for each prevented death.

Borrowing the language of mathematics and computability theory, undecidability derives from incommensurability, i.e., the impossibility of constructing a rule that leads to a correct yes-or-no answer. Thus, given the same initial conditions of scientific data, the final choice in conducting or not conducting screening is an ethical, not a scientific, issue. Scientists and the public have equal rights and competence to express an opinion on this subject. Indeed, the most recent and increasingly shared solution to the problem has been to release the final decision (and the dilemma!) to women [28]. However, new problems arise from this new approach since women’s preference collides with uncertainty about the benefits and harms; moreover, the transfer of responsibility is a forcing of the informed choice and can cause trouble to women [31]. The Population-based Research Optimizing Screening through Personalized Regimens initiative has been promoted by the US National Cancer Institute: it is based both on patient preferences and on optimizing the personalized benefit-harm ratio associated with screening [32, 33].

Regarding the main topic of my discussion, many scientists seem to ignore this dilemma. Scientists disposed to PA support the intervention; those disposed to PB discourage it.

## Incommensurability between paradigms

### PA: Systems medicine the main representative

Systems medicine is an application in the medical field of systems biology [34], which developed at the beginning of this century [35]. Systems medicine is also termed P4 systems medicine (P4SM) because it claims to be Predictive, Preventive, Personalized, and Participatory. Predictive and Preventive are strictly interrelated items, as reported by [36]: “Moreover, P4 medicine will in the future be able to predict the potential future emergence of disease-perturbed networks in patients and then design ‘preventive drugs’ that will block the emergence of these disease-perturbed networks and their cognate diseases.” P4SM is Personalized: it considers each person as a unique individual and not as a statistical average. Therefore, each person is treated in a *personalized* way based on the big data collected during continuous monitoring. This intervention requires active *participation* and a positive contribution from the population; in this way, P4SM is able to provide actionable information, which it can use to improve health.

To achieve the P4 goals, P4SM has to integrate data from conventional sources (including paper patient records, clinical and pathological parameters, and molecular and genetic data) and new sources (originating from statistical, mathematical, and computational tools). The procedure requires teams that combine expertise from different disciplines as well as continuous spatial and temporal monitoring of every individual. That will allow the extrapolation, analysis, and relating of a huge amount of heterogeneous, structured, and unstructured data (big data) to determine the links between different phenomena and predict future ones.

To obtain a *prediction* regarding the status and behavior of a medically monitored person, it is necessary to proceed according to the following steps [2]. First, it is necessary to identify the system variables whose measurement and observation can be used to answer a particular question. Second, the interaction among those variables has to be characterized at the molecular, cellular, and whole-body level. Third, the consequences of such interaction have to be determined using a process whereby a complex system (the human body) may be assessed through reduced representation.

The consensus related to this new approach to medicine and its dissemination are highlighted by a number of international institutions working in the field. Examples include the Institute for Systems Biology in Seattle, United States [37], the European Institute for Systems Biology and Medicine [38], and the Suzhou Institute of Systems Medicine, China [39].

P4SM is the only international organized movement for the PA model. However, at the international level, PB is represented by numerous approaches, movements, and campaigns, often connected with one another. The PB approaches include the following: choosing wisely, watchful waiting, the Too Much Medicine campaign, slow medicine, complaints against overdiagnosis, and quaternary prevention. I hope that my list is fully comprehensive, and I present below a short summary of each one.

### PB: Approaches and movements

#### Choosing wisely

Choosing wisely is probably the most widespread and structured movement featuring PB. Its mission is to promote dialogue between clinicians and patients by helping patients choose care that is as follows: supported by evidence; not duplicative of other tests or procedures already received; free from harm; truly necessary [40]. The core idea is to reduce overutilization of inappropriate and essentially harmful tests, treatment, and procedures. This movement is known for having launched in 2012 a campaign that invites all medical specialty societies to develop a list of five tests and procedures that physicians and patients should question [41].

#### Watchful waiting

Watchful waiting is not an organized movement: it is an approach to health problems. It is an alternative to more aggressive treatment, whereby time is permitted to pass before applying a medical intervention or therapy, and it makes use of patient involvement [42]. Prostate cancer has received considerable attention in this regard [43]. This approach is also promoted by the National Cancer Institute, which in its *Dictionary of Cancer Terms* describe the concept of watchful waiting as follows: “Closely watching a patient’s condition but not giving treatment unless symptoms appear or change. Watchful waiting is sometimes used in conditions that progress slowly. It is also used when the risks of treatment are greater than the possible benefits” [44].

#### Too much medicine campaign

At the end of the past century, a long debate began with *The BMJ* on the theme of “too much medicine?” [45]. The campaign later became formalized and has a dedicated Web site [46], which specifies the following: “*The BMJ*’s Too Much Medicine initiative aims to highlight the threat to human health posed by overdiagnosis and the waste of resources on unnecessary care. We are part of a movement of doctors, researchers, patients, and policymakers who want to describe, raise awareness of, and find solutions to the problem of too much medicine*.*”

#### Slow medicine

The organized movement of slow medicine began in Italy in May 2011. The background to the movement is respect for nature and the environment, a sense of justice, and an aversion to waste and consumerism [47]. These ideas are shared with those of another movement, Slow Food, with whom Slow medicine undertakes contact and collaboration. In the declaration of the slow medicine movement, seven “poisons” of fast medicine are described. Collaboration of slow medicine with choosing wisely came about with the creation of Choosing Wisely Italy [48].

#### Complaints against overdiagnosis

Overdiagnosis and overtreatment are the most insidious consequences of overuse of medical interventions [49]. Therefore, all the studies and authors that denounce it completely belong to PB. Here, the topics are not new; however, they have gained international resonance in recent years as mathematical models have been used to calculate the magnitude of overdiagnosis and overtreatment. Pathirana et al. [50] identified five drivers of overdiagnosis: culture; health systems; industry; professionals; and patients/public.

#### Quaternary prevention

This approach to medicine has been formalized in the concept of “quaternary prevention,” and it has particularly developed in the field of general practice [51]. Indeed the concept of quaternary prevention appears in the *WONCA International Dictionary for General/Family Practice*, where it is defined as “Action taken to identify patient at risk of overmedicalization, to protect him from new medical invasion, and to suggest to him interventions, which are ethically acceptable” [52].

I now present a comparison between PA and PB. First, I analyze their sensitivity and specificity; then, I assess their reduction to a harm-benefit ratio.

### Sensitivity and specificity of the two paradigms

As systems medicine, PA pursues the goal of monitoring (in a continuous spatial and temporal modality) all individuals. Therefore, owing to this putative ability to predict and prevent each future disease, PA has a very high sensitivity: theoretically, almost 100% for any clinical condition. However, the specificity and positive predictive value are supposedly low, depending on such factors as the disease, its prevalence, and the threshold used. By contrast, PB acts with restraint and when there is certainty of a favorable balance in terms of harms and benefits. Thus, for each disease, PB has a high specificity and high positive predictive value: only a small part of the population will be diagnosed and treated, i.e., individuals who are more probably ill or for whom interventions are more appropriate. Obviously, this approach has low sensitivity, which entails the risk of losing sick people. A well-known ancient ethical dilemma, applied to single clinical problems (as I saw previously with breast cancer screening), here applies to the whole population: to include all sick people at the cost of treating many both overdiagnosed and healthy individuals (overdiagnosis, overtreatment), or not to include all sick people (underdiagnosis, undertreatment) with the advantage of not treating healthy individuals.

### Reduction of comparison between PA and PB in harm-benefit assessment

Translating these considerations to the field of harm-benefit assessment, PA and PB have different approaches. In brief, PA entails high sensitivity, more benefits, and more harms; PB entails high specificity, fewer harms, and fewer benefits. Let us imagine ideal conditions under which both PA and PB offer the better performance. PA attempts to obtain maximum benefits for the population at the cost of increasing the damage somewhat (more benefits and a few more harms). PB tries to achieve minimum harms for the population at the cost of decreasing the benefits somewhat (fewer harms and slightly reduced benefits). This is the kind of incommensurability that I intend to address in comparing PA and PB: it is the impossibility of making a correct decision starting from these ideal conditions (undecidability). In my opinion, these are the core issues that arise from such a comparison. Therefore, the questions “Which is better?” and “Who is right?” are much too simplistic, or even nonsensical.

## Epistemological and ethical issues for PA

Thus, far I have focused on a comparison mainly of health aspects. If I broaden the horizon, PA, unlike PB, has epistemological contradictions and ethical troubles; PB involves potential harm for the population.

From an epistemological perspective, P4SM aims to achieve a shift in medicine from a reductionist approach to a holistic one: its tools take into account the complexity of the human body [53]. The main contradiction with this stance is that the complexity (in which the whole is greater than the sum of its parts) and holism are epistemologically incompatible with the type of predictivity claimed by P4SM. Thus, its flaunted predictive capacity could be an illusion. Moreover, I perceive the predictivity of P4SM as a return to the strictly deterministic approach to nature and to the human being. That is, if I consider each person a microcosm, it recalls the old declaration of Laplace’s demon [54]: if a demon knows the precise location and momentum of every atom in the universe, their future values for any given time are entailed. In terms of P4SM, this amounts to the following: if big data knows the precise location and momentum of every biological parameter for each individual, that person’s future values for any given time are entailed. In conclusion, I believe that P4SM is not a paradigm change, as suggested in [55]; it is an extreme form of the current PA and that Vogt et al. [56] define as a “technoscientific holism.”

From an ethical viewpoint, several criticisms have been made. Health anxiety [57] and cyberchondria [58], the modern versions of old hypochondria, have appeared and are a consequence of the current trend of medicine becoming increasingly invasive. The boomerang effect is that people are not feeling healthier but are constantly anxious about their health. These conditions could increase with the spread of systems medicine: it could become widely invasive of human life, and it has implications of social and cultural iatrogenesis up to the medicalization of health and life itself [56].

Other AA. underline the risks to individuals in the circulation of uncovered negative or potentially discriminatory health-related findings [59]. Mittelstadt and Floridi [60] consider big data particularly challenging from an ethical perspective owing to the sensitivity of health data and the fiduciary nature of health care; they identify 11 ethical risks connected to the spread of big data.

Further difficulties arise from the compatibility of predictive decisions derived from big data with the principles of clinical practice and evidence-based medicine. This is because systems medicine is a top-down model, providing clinicians with a computer algorithm; it is not based on clinical evidence and research [61].

Despite these limits, the words of the advocates of systems medicine are attractive and astonishing because they promise to free humanity from the burden of disease: “Regular check-ups will allow the physician to longitudinally follow each patient and detect any perturbation that might lead to disease long before the onset of disease symptoms. In this manner, an individual’s wellness can be preserved without the disease state ever occurring” [62].

## Conclusions

What lessons can be drawn from analyzing PA and PB if—beyond their intentions—neither PA nor PB can keep their promise of better health? What do we want from the medicine of the future: greater benefits but greater harm, or fewer harms and fewer benefits? Different scenarios are possible.

### Shift to PA

Owing to the ineluctable evolution of science and medicine, PA, as systems medicine, could become increasingly accessible and widespread in the population. Some or many countries will include systems medicine in their national health services; accordingly, many people or the whole population under such health services will be treated in that way. Therefore, PA could steadily become the dominant paradigm and PB could become marginal or disappear.

### Shift to PB

Over time, the promises of systems medicine are not maintained; that is because ethical problems arise, risks and harms are recognized, and the power of big data prediction is not realized. Furthermore, if such promises were partially or totally realized, the management of all that information would still be difficult, and the costs would become unsustainable. Together with the downsizing of PA, the concepts of overdiagnosis and overtreatment would become established and spread to the population. Thus, demand for PB would become dominant, and medicine would be practiced in a more contained, sober, and sustainable manner. PA could disappear or maintain a niche of activities for a few health obsessives, who voluntarily wish to undergo continuous monitoring. This development would be favored by a change in the world capitalist structure, with a downsizing of the dominant neoliberalism that is the cultural ground on which P4SM develops.

### Simultaneous presence of PA and PB

If the debate between the two paradigms is intrinsically undecidable because it is impossible to construct adequate decision making, then the debate could never end. Therefore, this scenario constitutes the persistence over time of the equal presence in epistemology and health policies of these two visions of medicine. PB could gain more support, continuing to counteract the excesses of PA and declaring the risks of too much medicine. PA could be available, free or at a cost, for people who wish to undergo it.

### Harm-benefit assessment between PA and PB

The choice between PA and PB refers essentially to a harm-benefit ratio. Thus, in the future it will perhaps be possible to overcome the incommensurability between them using the same big data as PA and performing a kind of meta-analysis harm-benefit analysis comparing PA and PB for all health problems and for all the population: *ex ante*, that could be achieved using sophisticated mathematical models; *ex post*, the analysis could compare populations submitted to P4SM with those not submitted to it. Moreover, it is also likely that in any scenario, the role of patients will become increasingly decisive.

In conclusion, the future attitude of medicine and health systems will depend not only on the ability to demonstrate the best health outcomes but also on a complex intertwining of political, economic, social, and cultural factors.

## Abbreviations

P4SM: P4 systems medicine
PA: Paradigm A
PB: Paradigm B
